# Application of family-centered empowerment model in primary caregivers of premature infants: A quasi-experimental study

**DOI:** 10.3389/fped.2023.1137188

**Published:** 2023-04-17

**Authors:** Kun Dai, Xinqi Fan, Huan Shi, Xiaoju Xiong, Lingli Ding, Yaqi Yu, Genzhen Yu, Suqing Wang

**Affiliations:** ^1^ Department of Public Health, Wuhan University, Wuhan, Hubei, China; ^2^ Department of Neonatology, Tongji Hospital, Tongji Medical College, Huazhong University of Science and Technology, Wuhan, Hubei, China; ^3^ School of Nursing, Tongji Medical College, Huazhong University of Science and Technology. Wuhan, Hubei, China

**Keywords:** family-centered education empowerment model, premature infants, primary caregiver, ability to care, readiness for hospital discharge

## Abstract

**Objective:**

To explore the effect of the family-centered empowerment model (FECM) on reducing anxiety, improving care ability, and readiness for hospital discharge of main caregivers of preterm infants.

**Methods:**

The primary caregivers of preterm infants who were admitted to the Neonatal intensive care Unit (NICU) of our center from September 2021 to April 2022 were selected as the research objects. According to the wishes of the primary caregivers of preterm infants, they were divided into group A (FECM group) and group B (non-FECM group). The intervention effects were evaluated with the Anxiety Screening Scale (GAD-7), the Readiness for Hospital Discharge Scale-Parent Version (RHDS-Parent Form), and the Primary Caregivers of Premature Infants Assessment of Care Ability Questionnaire.

**Results:**

Before the intervention, there was no statistically significant difference in the general information, anxiety screening, the scores of each dimension, and total score of the comprehensive ability of the main caregivers, and the score of caregiver preparedness between the two groups (*P* > 0.05). After the intervention, there were statistically significant differences in the anxiety screening, the total score and total score of each dimension of the care ability, and the score of caregiver preparedness between the two groups (*P* < 0.05).

**Conclusions:**

FECM can effectively reduce the anxiety of primary caregivers of premature infants and improve their readiness for hospital discharge and care ability. To improve the quality of life of premature infants by implementing personalized training, care guidance, and peer support.

## 1. Introduction

At present, about 70% of perinatal diseases in neonatal wards worldwide occur in preterm infants (1). Preterm infants are at high risk of abnormal development, and effective intervention should be carried out as soon as possible to improve the quality of life of preterm infants (2).

Most preterm infants are admitted to the neonatal intensive care unit (NICU) after birth. After the child is admitted to the ICU, the whole family will go through a period of grief, especially the mother, who will show great pain and often develop post-traumatic stress disorder (3–5). At the same time, the stress and uncertainty of neonatal admission to the NICU may persist after discharge and may contribute to the persistence of parental symptoms of depression and anxiety (6). Ensure that discharged preterm infants receive adequate care and stay healthy outside the hospital, how to improve the ability of family members to take care of their infants independently, distinguish and deal with abnormal conditions, and making them feel ready for discharge is a great challenge for all medical staff (7). Therefore, how to closely link doctors and nurses together to participate in the comprehensive nursing model is worthy of in-depth study.

Family-centered empowerment education models can enhance the family system, that is, empower the patient and other family members to improve the patient's health, and prevent diseases and complications (8, 9). The implementation of the model was divided into perceived problem-solving, educational engagement, and assessment.

Based on the above, the purpose of our study was to explore the effects of the family-centered empowerment model on anxiety, care ability, and discharge readiness of family main caregivers by taking primary caregivers of premature infants as the research object.

## 2. Materials and methods

### 2.1. Subjects and inclusion and exclusion criteria

**Figure 1:**
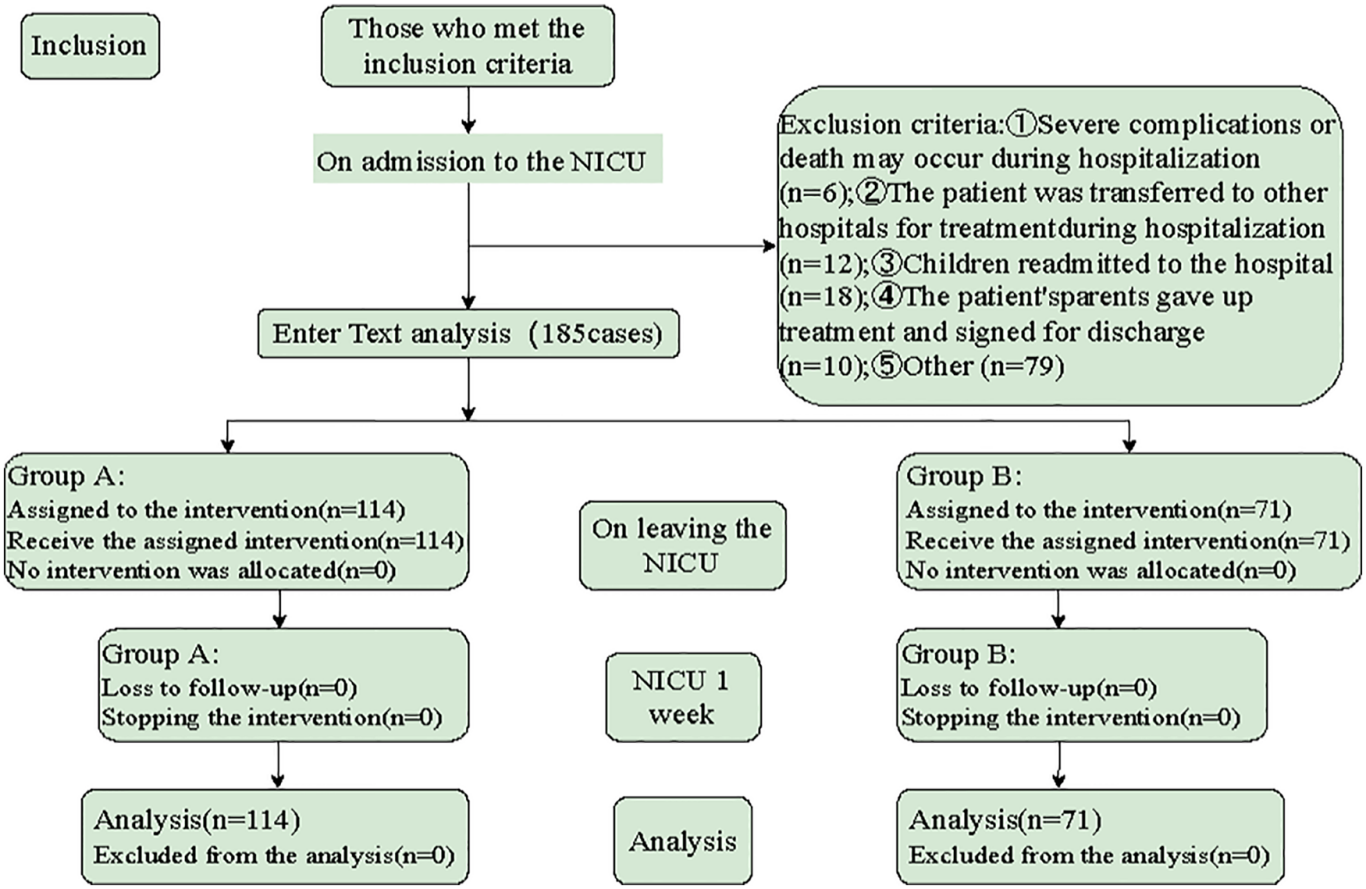
The selection process of research subjects.

In this quasi-experimental study, the main caregivers of premature infants admitted to the NICU of our center from September 2021 to April 2022 were selected as the research objects. The inclusion criteria were: ① Gestational age of premature infants <37 weeks; ② The weight of premature infants >1,800 g; ③ The vital signs of premature infants were stable 24 h; ④ The primary caregivers of premature infants had clear consciousness and basic ability to care for premature infants and read and understand. Exclusion criteria: ① Premature infants were transferred to other hospitals for treatment during hospitalization; ② premature infants who were readmitted; ③ Premature infants' family members gave up treatment and asked for signature to be discharged; The study objectives and process were explained to the participants, and anonymity and confidentiality of the information were guaranteed. According to the wishes of the main caregivers of preterm infants, they were divided into group A (FECM group) and group B (non-FECM group) (Figure 1).

### 2.2. Ethical considerations

Before the start of the study, the members of the FECM team explained the purpose, significance, and importance of the research process to the primary caregivers of preterm infants, obtained the support and cooperation of the primary caregivers, and signed the informed consent form.

### 2.3. Research methods

#### 2.3.1. Before intervention

A FECM team was established with professional and trained FECM team members to distribute questionnaires to parents and a psychiatrist as a consultant during questionnaire collection. The FECM team consisted of three medical directors (including the psychiatrist), two attending physicians, one chief nurse, three head nurses, three specialist nurses, and three graduate students. Doctors were mainly responsible for explaining the current condition of premature infants and disease-related knowledge, and other personnel was responsible for the formulation, evaluation, and review of health education manuals and family-centered empowerment education programs for main caregivers of premature infants.

#### 2.3.2. Intervention

##### 2.3.2.1. Intervention methods of group A (FECM group)

- Step 1: The perceived threat of illness, to assess and improve caregivers' knowledge of the severity of illness and the extent to which they felt threatened by the preterm infant's condition. After admission to the rooming-in room, ① “Instructions for Admission to the Ward” was distributed to the parents, and ward-related education was carried out. ② The head of the department and the bedside doctors explained to the main caregivers the current condition of the preterm infants, the problems that need attention, and the potential complications. ③ The main caregivers of premature infants who were admitted to the maternal-infant rooming-in set up a guardian meeting group with 4–8 people for 45–60 min, and the responsible nurses explained and distributed printed brothels, including the nature, etiology, and main clinical manifestations of the disease. ④ The primary nurses accompanied the primary caregivers to fill in the relevant questionnaires and scales. ⑤ Psychological counseling was given to the main caregivers.
- Step 2: Solve the problem, solve the present, set up a baby refueling class to teach the primary caregivers basic skills to manage the disease and its symptoms, and collect feedback intensively after class. The class time was 45–60 min, and the feedback was concentrated for 20 min after class. Classroom teaching mainly included basic care skills, feeding and nutrition, development support and interaction, observation of symptoms and signs, first aid knowledge, safety precautions, special care, and support outside the hospital. The focused feedback after class mainly included sharing, learning, and later care goals of the main caregivers. After-class focused feedback consisted of assessing the knowledge gained from the previous session by asking the patient 2–3 questions. In this step, these issues are addressed through group studies aimed at improving the self-efficacy, self-esteem, and self-control of the students.
- Step 3: Educational participation, through group discussions with 4–8 people for 60–80 min under the guidance of the primary nurse, allows the primary caregiver to face their problems and the problem-solving process, discuss their current situation and what they have done to solve similar problems, At the same time, according to the care ability improvement plan of the main caregivers of premature infants and the feedback of the main caregivers after class, the care level was evaluated, and finally, a practical solution was reached.
- Step 4: Assessment, formative assessment, and summative assessment. The formative assessment aimed to stimulate primary caregivers to internalize their sources of control by understanding their self-empowerment, which emphasizes self-responsibility for the premature infant's health. The effects of the intervention on the caregivers' anxiety (GAD-7 scale), readiness for hospital discharge (RHDS-Parent Form), and parenting competence (Preterm Infant Primary Caregiver Assessment of Caregiving Competence Questionnaire) were assessed in a summative manner. The discharge support system was established on the day of discharge, the discharge follow-up group was established, and the WeChat of FECM team members was added to solve the problems of discharged patients in time. Telephone follow-up was conducted 3 days, 1 week, and 1 month after discharge to ask the main caregivers about the current parenting problems and informed the solutions.

##### 2.3.2.2. Intervention methods of group B (non-FECM group)

The premature infants were treated and cared for by medical staff throughout the hospital. The main caregivers were discharged according to the routine after receiving the discharge notice, and the responsible nurses informed the relevant precautions of home care. On the day of discharge, they received routine discharge education, distributed education manuals, informed the precautions, and informed the main caregivers to conduct telephone follow-ups 3 days, 1 week, and 1 month after discharge.

### 2.4. Research tools

① General information questionnaire: including information on children and main caregivers. The children's information included name, gender, etc., and the main caregivers' general information included education level, age, etc. ② GAD-7: This scale was designed by Spitzer et al. (10) and other scholars according to the diagnostic criteria of the fourth edition of the American Diagnostic and Statistical Manual of Mental Disorders (DSM-IV). It adopts the 4-level scoring method containing 7 items. The higher the score, the higher the degree of anxiety. ③ RHDS-Parent Form: The RHDS-Parent Form was compiled by Wiess (11) according to the transition theory of Meleis in 2006, including 5 dimensions and 31 items, including 2 questions and 29 items. ④ The primary caregivers of premature infants' care ability assessment questionnaire: the questionnaire was self-designed, was divided into 8 parts, and had a total of 47 items. After the researchers' test, Cronbach's *α* coefficient of the questionnaire was 0.97. Eight experts were invited to evaluate the content validity of the questionnaire, and the content validity was 0.95, which showed high reliability and validity, and could be used in clinical research.

### 2.5. Statistical analysis

SPSS21.0 software was used for data analysis. The measurement data were described by $\bar{x}\pm S$ and compared by *t*-test. Enumeration data were described by examples and percentages and compared by the *χ*^2^ test. Ordinal data were described by cases and percentages and compared by a non-parametric test (Kruskal-Wallis *H* test). The anxiety and depression Scale of the main caregivers at discharge from NICU, the discharge readiness scale of the main caregivers at 1 week after discharge from NICU, and the care ability of the main caregivers at 1 week after discharge from NICU were compared between different nursing models. Multivariate linear regression analysis was used for multivariate analysis, with *α *= 0.05 as the statistical test level. *P* < 0.05 was considered statistically significant.

## 3. Results

### 3.1. Comparison of general information

In the comparison of general information between the two groups, except for the item of “whether they have received knowledge about premature infants”, the difference was statistically significant (*P* < 0.05), there was no significant difference in other items (*P* > 0.05), and the baseline was comparable, as shown in Table 1.

**Table 1:** Comparison of general information.

The project	Grade of classification	FECM Group (%)	Non-FECM Group (%)	Value of statistics	*P*
Gender	Male	63 (55.3)	108 (55.1)	0.001^a^	0.537
	Female	51 (44.7)	88 (44.9)		
Mode of delivery	Natural birth	23 (20.2)	45 (23.0)	0.568^a^	0.670
	Cesarean section	91 (79.8)	151 (77.0)		
Parity	Single birth	66 (57.9)	133 (67.9)	10.296^a^	0.006
	Twin twins	38 (33.3)	60 (30.6)		
	Triplets or more	10 (8.8)	3 (1.5)		
Have a brother, sister	No	68 (59.6)	125 (63.8)	0.470^a^	0.469
	Yes	46 (40.4)	71 (36.2)		
History of resuscitation at birth	Yes	27 (23.7)	43 (21.9)	0.142^a^	0.931
	No	51 (44.7)	91 (46.4)		
	Not clear	36 (31.6)	62 (31.6)		
Place of residence	Cities	84 (73.7)	134 (68.4)	1.401^a^	0.496
	Township	9 (7.9)	9 (11.7)		
	Rural areas	21 (18.4)	39 (19.9)		
Degree of education	Primary school and below	0 (0.0)	8 (4.1)	0.244^b^	0.621
	Secondary school or secondary school	43 (37.7)	58 (29.6)		
	College or bachelor's degree	62 (54.4)	112 (57.1)		
	Master's degree or above	9 (7.9)	18 (9.2)		
Monthly household income (Yuan)	<2,000	9 (7.9)	11 (5.6)	0.649^b^	0.421
	2,000–5,999	49 (43.0)	82 (41.8)		
	6,000–10,000	35 (30.7)	61 (31.1)		
	>10,000	21 (18.4)	42 (21.4)		
Status of work	On the job	77 (67.5)	132 (67.3)	0.055^a^	0.973
	Farmer	4 (3.5)	6 (3.1)		
	Out of work	33 (28.9)	58 (29.6)		
Experience in caring for premature infants	Yes	15 (13.2)	33 (16.8)	0.745^a^	0.420
	No	99 (86.8)	163 (83.2)		
Whether they have received knowledge about premature infants	Yes	18 (15.8)	53 (27.0)	5.167^a^	0.023
	No	96 (84.2)	143 (73.0)		

a. Chi-square test; b. Nonparametric test (Kruskal-Wallis H test)

### 3.2. Comparison of GAD-7

The results of this study showed that there was no statistically significant difference in the total score of the anxiety screening scale of the main caregivers of the two groups at admission and discharge (*P* > 0.05). One week after discharge, the total score of the anxiety Screening scale of the main caregivers in the two groups was statistically significant (*P* < 0.05), as shown in Table 2.

**Table 2:** The scores of the self-rating anxiety scale (GAD-7) of the main caregivers of premature infants in the two groups at discharge and 1 week after discharge.

Dimension	On Admission to the NICU				On leaving the NICU				It was NICU for 1 week			
	FECM Group (*n* = 114)	Non-FECM Group (*n* = 196)	*t*	*P*	FECM Group (*n* = 114)	Non-FECM Group (*n* = 71)	*t*	*P*	FECM Group (*n* = 114)	Non-FECM Group (*n* = 71)	*t*	*P*
The total score on the Anxiety Scale	7.60 ± 6.07	6.46 ± 5.57	1.660	0.090	6.46 ± 5.54	6.01 ± 5.21	0.551	0.582	5.76 ± 4.59	7.25 ± 5.33	−2.017	0.045

### 3.3. Comparison of RHDS-parents form

Comparison of RHDS of primary caregivers of premature infants between two groups: The results of this study showed that there was no statistically significant difference in the total score of RHDS of primary caregivers of two groups at discharge (*P* > 0.05). One week after discharge, the total scores of RHDS of the main caregivers in the two groups were statistically significant (*P* < 0.05), as shown in Table 3.

**Table 3:** The scores of RHDS-parents of primary caregivers of premature infants in the two groups at discharge and 1 week after discharge.

Dimension	On leaving the NICU				It was NICU for 1 week			
	FECM Group	Non-FECM Group	*t*	*P*	FECM Group	Non-FECM Group	*t*	*P*
Parents’ condition	50.54 ± 8.85	46.72 ± 10.65	2.641	0.009	48.94 ± 5.23	46.10 ± 6.45	3.279	0.001
The child's condition	40.89 ± 9.01	39.73 ± 10.81	0.790	0.431	44.09 ± 5.75	41.39 ± 7.03	2.841	0.005
Knowledge of disease	52.16 ± 20.64	54.39 ± 20.01	−0.725	0.469	67.10 ± 11.70	58.31 ± 15.78	4.336	<0.001
Coping ability after discharge	20.86 ± 7.09	20.90 ± 6.38	−0.040	0.968	23.19 ± 4.08	20.83 ± 5.19	3.444	0.001
Expected Social support	32.76 ± 7.30	29.23 ± 8.81	2.958	0.003	32.64 ± 5.61	28.72 ± 6.50	4.351	<0.001
Total readiness score	197.22 ± 43.96	193.20 ± 49.80	0.575	0.566	215.96 ± 25.64	89.86 ± 26.80	31.960	<0.001

### 3.4. Comparison of self-assessment ability questionnaires

The results of this study showed that there was no statistically significant difference in the total score of the self-assessment ability questionnaire of primary caregivers of the two groups at discharge (*P* > 0.05). One week after discharge, the total scores of the self-assessment ability questionnaire of the main caregivers in the two groups were statistically significant (*P* < 0.05), as shown in Table 4.

**Table 4:** The scores of the care ability questionnaire of the main caregivers of premature infants in the two groups at discharge and 1 week after discharge.

Dimension	On leaving the NICU				It was NICU for 1 week			
	FECM Group	Non-FECM Group	*t*	*P*	FECM Group	Non-FECM Group	*t*	*P*
Basic care skills	12.97 ± 7.68	14.92 ± 8.89	−1.911	0.58	33.87 ± 3.25	25.72 ± 9.55	−1.814	0.071
Nutrition at feeding	14.58 ± 4.79	14.20 ± 4.90	0.332	0.741	61.69 ± 10.90	49.77 ± 14.83	6.279	<0.001
Developmental support and interaction	8.18 ± 5.70	8.04 ± 3.20	0.180	0.857	33.87 ± 3.25	25.72 ± 9.55	8.380	<0.001
The symptoms and signs were observed	20.86 ± 12.65	20.78 ± 7.61	−0.008	0.993	82.91 ± 8.77	64.80 ± 22.93	7.596	<0.001
First aid knowledge	8.98 ± 6.61	11.37 ± 5.28	−2.571	0.011	42.18 ± 4.50	33.48 ± 13.98	6.161	<0.001
Safety precautions	8.79 ± 5.36	7.73 ± 3.13	1.510	0.133	34.26 ± 3.15	27.68 ± 9.34	6.932	<0.001
Special care	9.36 ± 6.66	8.90 ± 4.41	0.514	0.608	49.50 ± 5.86	25.211 ± 10.09	20.710	<0.001
Parent-child relationship	3.92 ± 2.65	3.83 ± 2.02	0.245	0.807	17.25 ± 1.64	12.80 ± 5.30	8.339	<0.001
The total score of self-evaluation ability	87.64 ± 38.65	89.86 ± 26.80	−0.424	0.672	375.72 ± 27.34	295.93 ± 85.80	9.218	<0.001

### 3.5. Linear regression analysis

All the indicators (nursing mode, gender, delivery mode, parity, elder brothers, elder sisters, birth weight, rescue history at birth, the residence of main caregivers, age of main caregivers, education level, family monthly income, working status, the experience of caring for premature infants, whether receiving related knowledge of premature infants, Anxiety and Depression Scale scores at NICU admission, Score on the Readiness for Hospital Discharge Scale at NICU discharge, Score on the ability to care at discharge from the NICU) were compared The discharge readiness of the main caregivers of premature infants at NICU and the care ability of the main caregivers at discharge from NICU were included in the multiple linear regression analysis. The results showed that the nursing model and whether they had received the related knowledge of premature infants were the main influencing factors of anxiety and depression of the main caregivers of premature infants at discharge from NICU (*P* < 0.05), as shown in Tables 5, 6. In the linear regression analysis of the discharge readiness of the main caregivers of premature infants at 1 week after NICU, it was found that the discharge readiness of the main caregivers of premature infants in NICU was the main influencing factor (*P* < 0.01), as shown in Table 7. In the linear regression analysis of the primary care ability of premature infants 1 week after discharge from NICU, it was found that the nursing mode and the primary care ability of premature infants at discharge from NICU were the main influencing factors (*P* < 0.05), as shown in Table 8.

**Table 5:** Variable assignment table.

Variables of interest	Mode of assignment
Model of care	1 = FECM Group; 2 = Non-FECM Group
Gender	1 = Male; 2 = Female
Mode of delivery	1 = Natural birth; 2 = Cesarean section
Parity	1 = Single birth; 2 = Twin twins; 3 = Triplets or more
Have a brother, sister	1 = No; 2 = Yes
Weight at birth	Original value input
History of resuscitation at birth	1 = Yes; 2 = No; 3 = Not clear
Place of residence of the primary caregiver	1 = Cities; 2 = Township; 3 = Rural areas
Age of primary caregiver	Original value input
Education level of primary caregivers	1 = Primary school and below; 2 = Secondary school or secondary school; 3 = College or bachelor's degree; 4 = Master's degree or above
Monthly household income of the primary caregiver	1 = <2,000; 2 = 2,000–5,999; 3 = 6,000–10,000; 4 > 10,000
Experience in caring for premature infants	1 = On the job; 2 = Farmer; 3 = Out of work
Whether they have received knowledge about premature infants	1 = Yes; 2 = No
Whether they have received knowledge about premature infants	1 = Yes; 2 = No

**Table 6:** Regression analysis of anxiety and depression in primary caregivers of premature infants at discharge from NICU.

Variables of interest	On leaving the NICU			
	*β*	95% CI	*t*	*P*
Grouping	0.477	4.418–7.365	−7.884	<0.001
Male	0.102	−0.216 to 2.757	1.685	0.094
Cesarean section	−0.013	−1.967 to 1.595	−0.206	0.837
Multiple births	−0.032	−1.680 to 1.026	−0.477	0.634
No elder brother or elder sister	−0.072	−2.577 to 0.789	−1.047	0.296
High birth weight	−0.009	−1.006 to 0.868	−0.146	0.884
There was no history of rescue at birth	0.082	−0.315 to 1.750	1.370	0.172
The place of residence was a city	−0.039	−1.351 to 0.732	−0.585	0.559
The caregivers were older	−0.005	−0.1580 to 0.145	−0.083	0.934
The education level of the caregivers was college or undergraduate	−0.044	−2.010 to 1.101	−0.576	0.565
The family monthly income of the caregivers was 6,000 to 10,000	0.035	−0.716 to 1.210	0.506	0.613
The working status of the main caregivers was on-the-job	−0.063	−1.335 to 0.476	−0.934	0.351
Experience in caring for premature infants	−0.090	−4.019 to 0.875	−1.266	0.207
Have received knowledge of premature infants	−0.154	−4.649 to −0.220	−2.168	0.031
The degree of anxiety and depression is high upon admission to NICU	0.048	−0.078 to 0.175	0.757	0.450

*R*^2 ^= 0.308; Adjusted *R*^2 ^= 0.260; *F* = 6.452, *P* < 0.01.

**Table 7:** Linear regression analysis of discharge readiness of primary caregivers of preterm infants at 1 week after NICU.

Variables of interest	NICU 1-week readiness for Hospital Discharge Scale was developed			
	*β*	95% CI	*t*	*P*
Grouping	10.502	−1.705 to 22.709	1.697	0.091
Male	−1.694	−13.839 to 10.452	−0.275	0.784
Cesarean section	−12.594	−26.882 to 1.694	−1.739	0.084
Multiple births	7.584	−3.561 to 18.730	1.343	0.181
No elder brother or elder sister	10.638	−3.383 to 24.658	1.497	0.136
High birth weight	3.060	−4.473 to 10.592	0.801	0.424
There was no history of rescue at birth	−1.365	−9.779 to 7.049	−0.320	0.749
The place of residence was a city	−2.128	−10.535 to 6.279	−0.499	0.618
The caregivers were older	0.092	−1.159 to 1.342	0.145	0.885
The education level of the caregivers was college or undergraduate	4.997	−7.812 to 17.806	0.770	0.442
The family monthly income of the caregivers was 6,000 to 10,000	4.104	−3.889 to 12.098	1.013	0.312
The working status of the main caregivers was on-the-job	0.485	−7.021 to 7.991	0.127	0.899
Experience in caring for premature infants	10.183	−11.232 to 31.598	0.938	0.349
Have received knowledge of premature infants	2.788	−16.858 to 22.435	0.280	0.780
The readiness for hospital discharge was high at discharge from NICU	0.568	0.146–0.310	5.506	<0.01

*R*^2 ^= 0.210; Adjusted *R*^2 ^= 0.145; *F* = 3.257 *P* < 0.01.

**Table 8:** Results of linear regression analysis of care ability of primary caregivers of premature infants at 1 week after NICU.

Variables of interest	The NICU 1 week care ability assessment questionnaire was used			
	*β*	95% CI	*t*	*P*
Grouping	233.999	214.016–253.983	23.101	<0.01
Male	5.106	−14.776 to 24.989	0.507	0.613
Cesarean section	−5.638	−29.028 to 17.752	−0.476	0.635
Multiple births	11.379	−6.867 to 29.624	1.230	0.220
No elder brother or elder sister	3.331	−19.621 to 26.283	0.286	0.775
High birth weight	−2.752	−15.082 to 9.579	−0.440	0.660
There was no history of rescue at birth	−0.592	−14.366 to 13.182	−0.085	0.932
The place of residence was a city	13.987	0.224–27.749	2.005	0.046
The caregivers were older	1.232	−0.815 to 3.278	1.187	0.237
The education level of the caregivers was college or undergraduate	29.835	8.867–50.803	2.807	0.006
The family monthly income of the caregivers was 6,000 to 10,000	−4.192	−17.278 to 8.893	−0.632	0.528
The working status of the main caregivers was on-the-job	2.991	−9.297 to 15.279	0.480	0.632
Experience in caring for premature infants	13.432	−21.625 to 48.489	0.756	0.451
Have received knowledge of premature infants	2.118	−30.044 to 34.279	0.130	0.897
High care ability when leaving the NICU	0.130	0.029–0.272	2.444	0.015

*R*^2 ^= 0.767; Adjusted *R*^2 ^= 0.748; *F* = 0.303, *P* < 0.01.

## 4. Discussion

**Figure 2:**
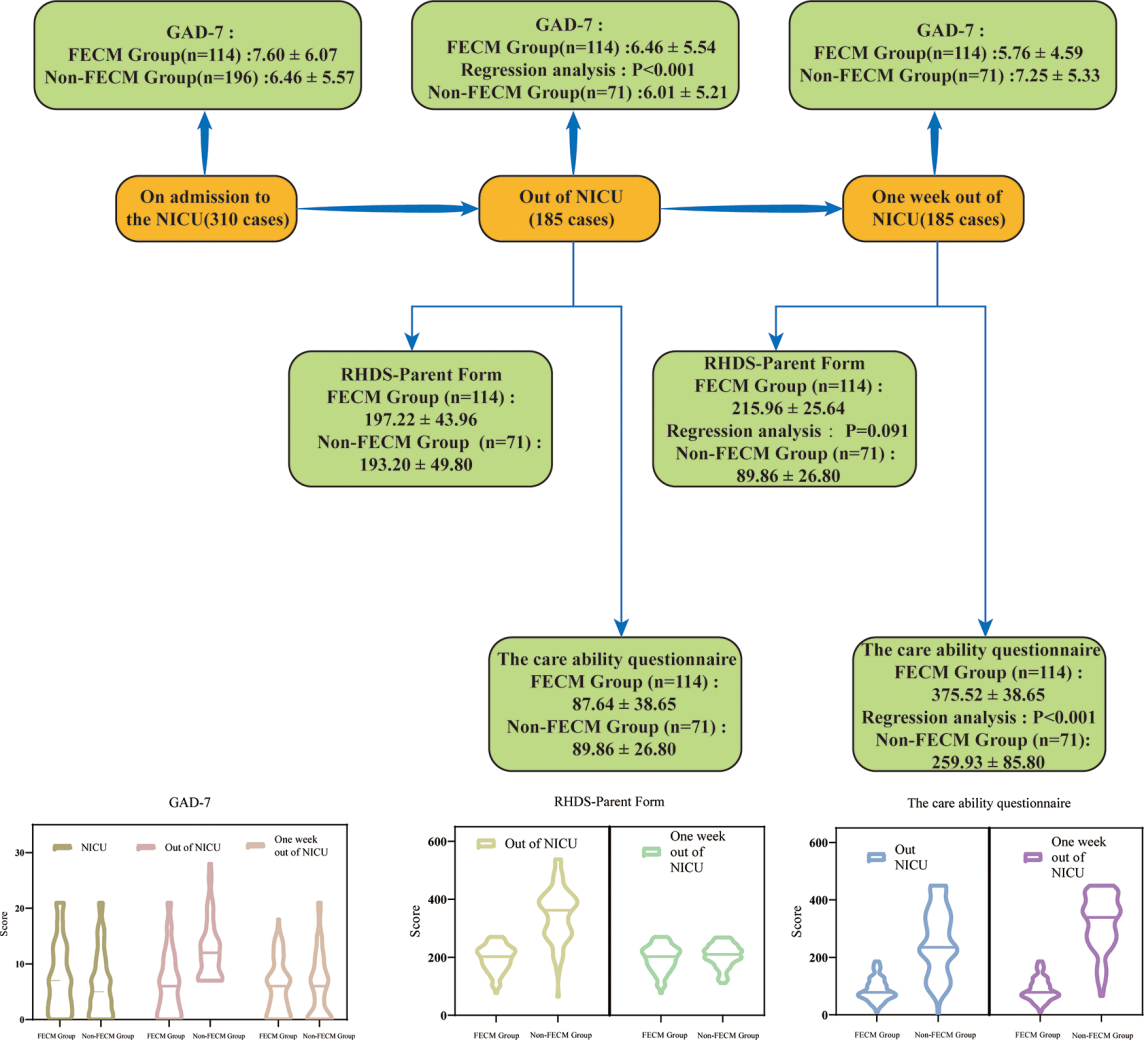
A summary figure of the study.

In this study, we found that a family-centered empowerment model not only reduced the anxiety level of primary caregivers of preterm infants but also improved the readiness and caregiving capacity of primary caregivers of preterm infants. The overall results can be seen in Figure 2.

FEMC intervention significantly reduced the anxiety of primary caregivers of premature infants: The results of this study showed that there was no significant difference in the scores of primary caregivers between the two groups at baseline (*t* = 1.660, *P* > 0.05). Before the intervention, the anxiety scores of primary caregivers in the two groups were higher, and there was no significant difference in the scores between the two groups (*t* = 0.551, *P* > 0.05). After the intervention, the score of group A (FECM group) was significantly lower than that of group B (non-FECM group) (*t* = −2.017, *P* < 0.05). This indicated that the FECM intervention significantly reduced the anxiety of the primary caregivers. The results of the present study are similar to those of Shahdadi et al. (12), but while Shahdadi focused on hemodialysis patients, the present study applied FECM to primary caregivers of preterm infants. Through the implementation of FECM, this study changed the traditional way of main caregivers caring for premature infants and encouraged the main caregivers to become the leaders of nursing premature infants. Under the guidance of the FECM group, the main caregivers actively expressed their care needs, and formulated personalized care plans through group discussion, to reduce the psychological burden of the main caregivers and lay the foundation for the smooth discharge of late premature infants. In the process of caring for premature infants, due to the lack of corresponding experience and training, most of the main caregivers are extremely nervous, anxious, and at a loss. FECM intervention can alleviate the anxiety level of most of the main caregivers.

FECM intervention can significantly improve the discharge readiness of primary caregivers of premature infants: The results of this study showed that after the intervention, the total score of discharge readiness in the FECM group was higher than that before the intervention, and the total score of discharge readiness in the control group was lower than that in the intervention group (*t* = 31.960, *P* < 0.01). The FECM intervention could significantly improve the discharge readiness of the primary caregivers, indicating that only routine discharge education was given to the primary caregivers at discharge. The personalized home care needs of preterm infants cannot be met, which is similar to the results of Hulya's study (13). In this study, the FECM group took the care problems of the main caregivers as the starting point, paid attention to the psychological status of the main caregivers and provided peer support through group meetings, informed the main caregivers of feedback on the problems in the care of premature infants in time, and put forward personalized solutions. To provide effective care services to calmly and efficiently deal with the emergency of premature infants, and to improve the coping ability of primary caregivers after discharge. The results of Chen et al. also show that evaluating parents before discharge and helping them to master caregiving skills can facilitate a successful transition from hospital to home care and improve outcomes of preterm infants after discharge (14).

FECM intervention can significantly improve the care ability of the main caregivers of premature infants: The results of this study showed that there was no significant difference in the care ability of the main caregivers between the two groups before the intervention (*t* = −0.424, *P* > 0.05). FECM intervention significantly improved the care ability of the main caregivers (*t* = 31.960, *P* < 0.05). This result is similar to that of Masoodi et al. (15). Unlike Masood's research object, this study applied FECM to primary caregivers of premature infants to improve their comprehensive care ability. Through the training of the FECM group, this study advocates the main caregivers to actively participate in the baby oil classroom, group meetings, bedside skills training, and other projects, to maximize the rights of the main caregivers, cultivate the care skills of the main caregivers, improve the care ability of the main caregivers, and provide a prerequisite for a smooth transition to home care after discharge and improve the quality of life of premature infants. The results are similar to those of Raei et al. (16). At the same time, primary caregivers are not only providers of care services but also excavators of the potential abilities of preterm infants, which can significantly optimize the health outcomes of preterm infants (17). Therefore, medical staff should follow the intervention process of FECM to mobilize the enthusiasm of the primary caregivers and encourage them to participate in it, to improve the caring ability of primary caregivers.

However, this study still has some limitations. First, long-term follow-up after the intervention was lacking in this study, and further long-term follow-up can be carried out in the future to verify the long-term effects of FECM. Second, as this study is a single-center study, further multi-center prospective clinical studies are needed to prove the clinical validity of this model. However, our study is also very important clinically. The group meeting has achieved a good response in the implementation process, and peer support by the main caregivers of NICU can be used as an integral part of NICU service in later research.

## 5. Conclusions

The application of FECM in premature infants and their main caregivers can significantly reduce the anxiety of the main caregivers, improve the readiness for discharge and care ability, lay a solid foundation for the smooth transition from hospital care to home care, and improve the quality of life of premature infants through the implementation of personalized training, care guidance, and peer support.

## Data Availability

The original contributions presented in the study are included in the article/Supplementary Material, further inquiries can be directed to the corresponding author.
